# Diagnostic Accuracy of 3D Ultrasound and Artificial Intelligence for Detection of Pediatric Wrist Injuries

**DOI:** 10.3390/children8060431

**Published:** 2021-05-21

**Authors:** Jack Zhang, Naveenjyote Boora, Sarah Melendez, Abhilash Rakkunedeth Hareendranathan, Jacob Jaremko

**Affiliations:** Department of Radiology and Diagnostic Imaging, Walter C. Mackenzie Health Sciences Centre, University of Alberta, 8440-112 Street, Edmonton, AB T6G 2B7, Canada; jzzhang@ualberta.ca (J.Z.); naveenjy@ualberta.ca (N.B.); macdonel@ualberta.ca (S.M.); hareendr@ualberta.ca (A.R.H.)

**Keywords:** 3D ultrasonography, wrist, fractures, pediatric, artificial intelligence

## Abstract

Wrist trauma is common in children, typically requiring radiography for diagnosis and treatment planning. However, many children do not have fractures and are unnecessarily exposed to radiation. Ultrasound performed at bedside could detect fractures prior to radiography. Modern tools including three-dimensional ultrasound (3DUS) and artificial intelligence (AI) have not yet been applied to this task. Our purpose was to assess (1) feasibility, reliability, and accuracy of 3DUS for detection of pediatric wrist fractures, and (2) accuracy of automated fracture detection via AI from 3DUS sweeps. Children presenting to an emergency department with unilateral upper extremity injury to the wrist region were scanned on both the affected and unaffected limb. Radiographs of the symptomatic limb were obtained for comparison. Ultrasound scans were read by three individuals to determine reliability. An AI network was trained and compared against the human readers. Thirty participants were enrolled, resulting in scans from fifty-five wrists. Readers had a combined sensitivity of 1.00 and specificity of 0.90 for fractures. AI interpretation was indistinguishable from human interpretation, with all fractures detected in the test set of 36 images (sensitivity = 1.0). The high sensitivity of 3D ultrasound and automated AI ultrasound interpretation suggests that ultrasound could potentially rule out fractures in the emergency department.

## 1. Introduction

Fractures are the third leading cause of pediatric hospitalizations in Canada [1]. Distal radius fractures account for up to 25% of fractures documented in children [2]. Distal radius fractures typically occur in children falling on an outstretched hand and involve the metaphysis or physis [2]. Depending on the area of injury, there can be a multitude of fracture patterns that affect treatment planning [3]. Therefore, when children present to primary care clinics or emergency department (ED) with suspected wrist fractures, radiographs are the standard of care as they allow for precise examination of the anatomy. In most hospitals, routine radiographs are performed on patients with wrist trauma, but only half of the imaging reveals fractures [4]. With the estimated cost of treating pediatric forearm fractures at $2 billion per year in the USA [5], streamlining care is desirable. Obtaining radiographs in ED typically involves sending the patient to a separate diagnostic imaging area, where they wait in an additional queue, and transferring them back, a process which can add hours to an ED visit. If clinicians could determine at bedside who has a fracture and requires an X-ray, systemwide radiation doses and costs could be reduced and ED visits shortened.

Ultrasound (US) is a ubiquitous but underutilized tool in the ED. It has the advantages of being inexpensive and portable while being able to reveal cortical disruption, periosteal fluid, and joint effusion to aid in detecting fractures. The use of US in pediatric distal radius fractures has been validated to have similar accuracy compared to radiography [6,7,8,9,10,11]. A recent meta-analysis identified 16 studies with 1204 patients, resulting in 97% sensitivity and 95% specificity [12]. More recent research has shown that a short 1–2 h training session is sufficient for physicians to perform the US scan [7,9]. 3D ultrasound (3DUS), essentially a high-quality sweep video obtained across the injured area, should more fully depict the fracture anatomy than a single 2D ultrasound image, but there are currently very few studies examining the role of 3DUS in pediatric fracture detection.

In general, 3DUS interpretation involves reviewing large amounts of image data, and its accuracy depends largely on the clinician’s expertise. As a possible alternative to manual interpretation, we examined the feasibility of automatic interpretation of wrist ultrasound using AI. Our technique used an ensemble of convolutional neural networks (CNNs) that predicted the presence/absence of a fracture in a given image.

Accordingly, the aim of this study was to assess feasibility, reliability, and accuracy of 3DUS, interpreted by humans or automatically by AI, for detection of pediatric wrist fractures, in comparison to conventional radiographs. We hypothesized that these advanced tools allow use of ultrasound to detect fractures as accurately as radiographs.

## 2. Materials and Methods

### 2.1. Study Design

This was a prospective diagnostic study performed at a tertiary pediatric hospital. The study was approved by the institutional ethics committee (Pro00077093).

### 2.2. Study Protocol

A convenient sample of 30 children (age: 0–17 years) presenting to a tertiary pediatric hospital with unilateral upper extremity injuries to the wrist were identified at triage. Written informed consent was obtained from the parents (or the child if a mature minor > 16 years of age). Most US scans were performed in the waiting room prior to physician evaluation. Radiographs of the symptomatic limbs, as ordered by the EM physician at initial visit, and any follow-up imaging obtained over the next 30 days within our health region, were obtained from PACS, anonymized, and stored for blinded reading.

The inclusion criterion was tenderness in the distal radius region following trauma, such as falling on the arm. Exclusion criterion was inability to perform an ultrasound scan, such as due to an existing cast, laceration over scanning area, or open fractures. Children were not excluded based on severity of symptoms.

### 2.3. Imaging Technique and Training

Each child was seated, and their wrists were placed in a comfortable neutral position. Imaging was performed on a Philips IU22 machine using a 13 MHz 13VL5 probe for 3DUS. The child’s injured limb was scanned on the dorsal and volar surfaces in both the sagittal and axial orientation. The operator centered the view on the distal 3 cm of the radius for all children in the different orientations and initiated the sweep. A 3.2 second automated sweep through a range of ±15° to produce US slices of 0.2 mm thickness totaling 382 slices was performed. The non-injured wrists were similarly scanned. We thus had four sweeps of each wrist. The 3DUS probe was used to ensure consistent sweeps rather than imaging differences.

Training was deliberately kept minimal. The operators for scans were medical students (J.Z. and N.B.) with no prior experience with US. Each received 1 h of hands-on training by a pediatric MSK radiologist (J.J.) which consisted of primarily operating the IU22, a discussion on the basic anatomy of the distal forearm, and practice scanning the radiologist under supervision. Readers were given 5 examples of wrist fractures and 5 examples of non-fractured wrists for review prior to blinded reading.

### 2.4. Artificial Intelligence

CNNs have been gradually increasing in popularity for computer vision problems and are now the technique of choice due to success in image classification, as shown in competitions such as ImageNet [13]. CNN models consisting of convolutional layers and fully connected layers were trained to classify images frame-by-frame as either normal (category 0) or fractured (category 1). Convolutional layers are for detecting patterns, such as the edge of an uninjured bone, while fully connected layers interpret the detected patterns. For example, if the straight edge of a bone is suddenly disrupted, the fully connected layer might interpret that as a fracture. All frames were resized to match the network input size (128 × 128). Using a brute force approach, we trained several models with varying numbers of convolutional layers and fully connected layers and selected three networks that gave the highest accuracy to be part of the ensemble. The output of each ensemble represents the median prediction of the three CNNs included. The ranges of various network parameters used are summarized in Table 1. We trained separate ensembles for volar sagittal and dorsal sagittal scans.

### 2.5. Statistical Analysis

3DUS readings of both injured and uninjured limbs were anonymized and read independently by a medical student (J.Z.), pediatric radiology fellow (S.M.), and pediatric MSK radiologist (J.J.). The radiographs were centrally re-reviewed by our pediatric MSK radiologist blinded to clinical data and compared to original reports. Sensitivity (SN), specificity (SP), positive and negative predictive value (PPV, NPV), and positive and negative likelihood radio (LR+, LR−) were calculated for human experts and AI using SPSS (SPSS Inc., v.22, Chicago, IL, USA). Interrater reliability was calculated as percentage agreement and Cohen’s Kappa.

## 3. Results

We enrolled 30 children, resulting in 55 scans of individual limbs (five non-injured limbs could not be imaged due to logistical constraints in ED). The mean age was 9.9 years (range 3.8–14.8) and 70% of the participants were male. On radiographic review, unilateral arm radiographs of all 30 patients were assessed, revealing 19 distal forearm fractures. The blinded central re-review was in 100% agreement with original reports. Radiographic evidence of fracture was used as gold standard diagnosis.

### 3.1. Manual Interpretation

The diagnostic accuracy of 3DUS in identifying distal radius fractures in our dataset was 96.5% (95% CI 89–100%). Out of the 19 fractures, 19 were correctly identified by the radiologist and 18 were identified by the medical student and fellow. For limbs without fractures, we achieved an accuracy of 80% (95% Cl 63–98%). Out of the 36 uninjured arms, the radiologist had 6 false positives while the medical student and fellow had 5 and 10 false positives, respectively. Overall, the three readers performed similarly with moderate to high interrater reliability (Cohen’s kappa: J.J.–J.Z. 0.602, J.J.–S.M. 0.571, J.Z.–S.M. 0.536) [14]. By combining the three readers together, i.e., the diagnosis agreed upon by at least 2 readers, the combined reader had SN 1.00, SP 0.90 (Table 2).

**Figure 1:**
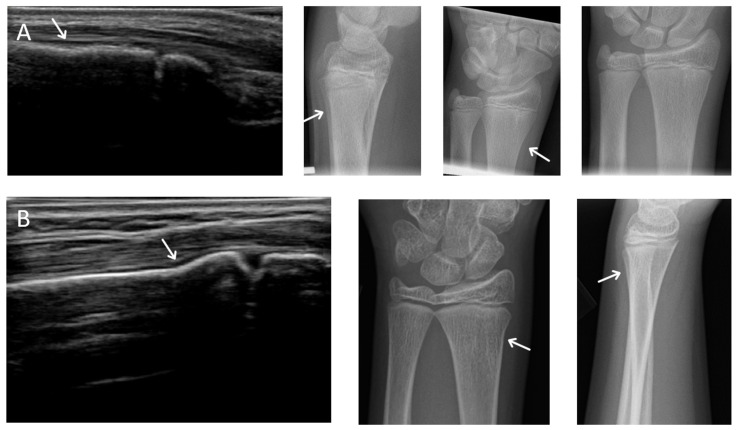
Possible missed fractures. (**A**,**B**) are dorsal longitudinal views of the distal radius of two possible X-ray false negatives. Note the excess angulation of the distal radius may represent torus fractures. The possible defect is observable on X-ray.

**Figure 2:**
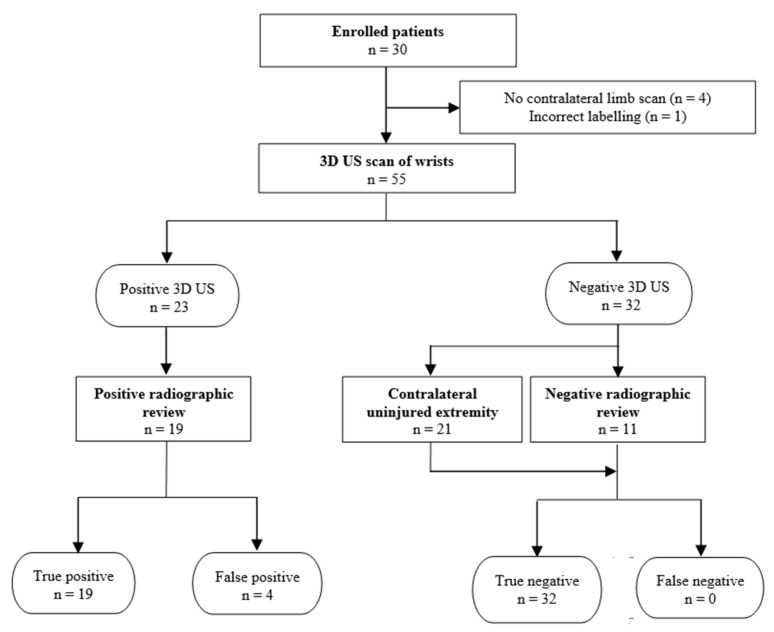
STARD flow diagram, showing the number of children who received the US and gold standard radiograph [15]. The US results are from combined readers.

The “combined reader” produced 4 false positives which were individually reviewed by the radiologist. Two scans were misclassified due to 3DUS artifacts and the other two scans represented possible subtle fractures that were not identified on radiographs (Figure 1). The combined reader did not have any false negatives (Figure 2).

### 3.2. AI Interpretation

We trained multiple CNN models using 2D images extracted from 21 wrist sweeps (~6000 images). We validated the AI prediction on 1640 image slices extracted from 72 3DUS volumes (36 volar and 36 dorsal) acquired from 36 wrists. Our technique gave accuracies of 95% and 94% per slice for images extracted from dorsal and volar views. Each 3DUS volume was analyzed slice by slice and sweeps with more than 30 slices predicted to be fractured were categorized as class 1. Our technique was 89% and 92% accurate in classifying dorsal and volar sweeps. For each patient, predictions on dorsal and volar sweeps were combined to arrive at a final AI-aided diagnosis. When compared against human assessments of the same patient, AI-aided diagnosis gave sensitivity = 100% and specificity = 89% (Table 3). Evaluation of agreement of the AI prediction with human readers gave ICC = 0.80 (CI (0.62, 0.92)) and Cohen’s Kappa = 0.48–0.74.

## 4. Discussion

In this pilot study, we found that 3D ultrasound was highly accurate at detecting fractures, whether interpreted by human experts or automatically by artificial intelligence.

A recent meta-analysis of 16 studies produced pooled statistics of 97% SN, 95% SP, 20 LR+, and 0.03 LR– for usage of 2D US in pediatric distal forearm fractures [12]. Our results are comparable to previous studies and demonstrate the ability of 3DUS to be a diagnostic tool in distal radius fractures. This is the first study to show that 3DUS scans allow radiologists and trainees to diagnose pediatric distal forearm fractures, with the benefit of minimal scanning and reading training.

The radiologist achieved very high sensitivity of 100% and 95% for the other 2 readers. While our data had a small number of fractures, it suggested that a negative 3DUS in ER can effectively rule out a distal radius fracture. A rational clinical management plan would be to send children with a positive 3DUS scan for radiographs to confirm the presence of a fracture and aid in treatment planning, while children with a negative 3DUS scan by this protocol could potentially be discharged without radiographs, reducing cost to the healthcare system. Although the trainees missed 5% of positive cases, none of the three readers had previous experience reading US images of wrist fractures and their training was limited to 5 fractures and 5 normal sweeps. Thus, due to limited experience, we combined the results from all three readers to offer insight into the performance of individuals if they had more training and experience. A larger study with more data for training and testing could improve overall performance. In addition, future studies investigating the ability of ultrasound in identification of the fracture pattern and treatment planning can further expand the clinical role of ultrasound. Future research involving ED physicians interpreting the wrist sweeps would determine whether these scans are useful in clinical practice.

Reviewing the discordant cases, there were several individual reader false positives, with the false-positive rate reduced by using the combined reading values, further suggesting the need for more training. Another reason for false positives was US artifacts, including double or interrupted cortical margins, likely caused by motion artifact and side-lobe artifact. The 3DUS obtained hundreds of images per sweep, and some of these images included artifacts that might not be identified as frequently on 2DUS. Adding 5 min of user training specifically demonstrating examples of these artifacts could help users avoid misinterpretation. Users who identify these artifacts at the time of scanning would be encouraged to perform repeat scans, subject to time constraints in the clinical department.

A benefit of 3DUS was that unlike in previous studies of 2DUS, 3DUS allowed retrospective review of the scans. Scans in previous studies were performed by emergency physicians or residents as part of the patient care team [7,10,16]. The history and physical exam alongside the US likely augmented the overall clinical picture and improved the SN and SP of those studies [10]. The 3D sweep allowed for a simple scanning protocol which reduced the training that medical students with limited clinical knowledge required to just 1 h. Training in previous studies focused on anatomy, identification of fracture, and viewing method [10,16]. We expect that ED physicians with point-of-care US training can readily employ this technique to evaluate patients and make decisions on additional imaging. Furthermore, while US images are considered highly user-dependent [9,17], by capturing a sweep of the fracture and allowing for retrospective review, user dependency can be reduced and allow any healthcare practitioner to obtain a useful scan, and ED physicians can review the scan at their convenience. This also allows radiologists to aid in the interpretation of the study if there are any concerns. With minimal user training, we replicated the high reported sensitivity of 2DUS using 3DUS. Our specificity with 3DUS was somewhat lower than the best reported 2DUS results, but on review of images, this could be rectified in the future by a few extra minutes of reader training. The value added by 3DUS was not in increasing the already high accuracy of well-performed 2DUS, but in allowing scans to be performed by users with less training, generating comprehensive saved images that can be reviewed more reliably.

**Figure 3:**
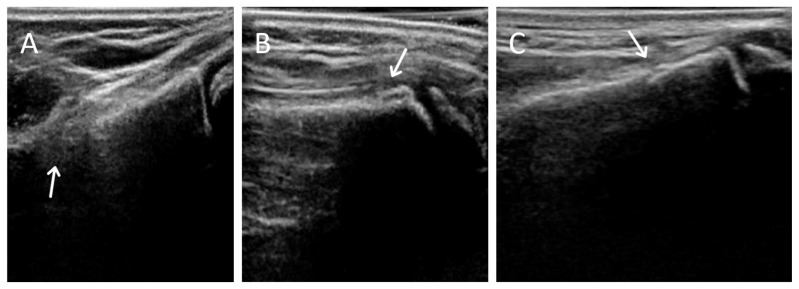
Examples of 3DUS scanning artifacts. (**A**) Edge effects, (**B**) double cortex from motion, (**C**) overlapping cortex.

There were 2 possible radiographic false negatives out of 10 negative radiographs, as the US revealed subtle cortical irregularities that could represent undisplaced fractures. Retrospective review of two X-rays in light of ultrasound findings showed subtle cortical contour irregularities that might have represented the fractures identified on US (Figure 3). Previous studies had also reported the possibility that ultrasound may identify subtle fractures not seen on radiographs [7]. The clinical significance of these possible undisplaced fractures is unknown. Future study could potentially perform limited MRI in this small subset of patients with discrepant ultrasound/X-ray findings to clarify vs. an external gold standard.

Another key benefit of 3DUS was that the ‘sweep’ images obtained gave a comprehensive dataset, showing the full anatomy of the distal radius and ulna, suitable for automated reconstruction using artificial intelligence (AI), and eventually, automated diagnosis of wrist fractures [17].

The 3DUS probe used in this study was costly but not necessary. We used 3DUS to ensure the sweeps produced were as consistent as possible for accurate training of the AI algorithm. Readily available conventional 2DUS probes can produce a cine ‘2D sweep’ video when the user moves any 2DUS probe manually across the arm. Our future research will include testing whether 2DUS manual ‘sweep’ videos can be interpreted with the same diagnostic accuracy as 3DUS sweeps. Ultimately, a package combining cine ultrasound acquisition by a minimally trained user using a portable handheld probe with automated AI analysis of the obtained images could be a rapid diagnostic aid to efficiently rule out or detect wrist fractures at the point of care.

### 4.1. AI Interpretation

A CNN-based technique was developed to automatically detect fractures by combining the information in volar and dorsal sagittal scans. It correctly detected all fractures, corresponding to expert-level sensitivity of 100% in 3D sweeps. One of 13 fractures was missed in the volar scans but correctly detected in the corresponding dorsal scan. This is expected as not all fractures involve the volar cortex. The model also showed high agreement with human interpretation in terms of ICC and kappa. Given that 3/13 fractures were missed by at least one human reader, the sensitivity of the AI technique (100%) is particularly high.

AI-aided analysis of 3DUS eliminates inter-observer variability and saves expert time. The end-to-end execution time of AI models on V100 NVIDIA GPU was <2 seconds per sweep, which is well-suited for real-time applications. The AI models can be accessed from emergency departments and the interpretation can be obtained along with the scan report.

### 4.2. Limitations

This study had limitations. We had a small sample size and limited the current study to the distal radius. Less common but important fractures of the distal ulna or scaphoid could potentially also be detected by 3DUS, and this requires further study. Due to ethical restrictions on radiation dose in children, we were unable to obtain radiographs of contralateral limbs. It was presumed that no acute fractures were present in these 30 limbs, all of which were asymptomatic (nontender and moved freely by the children during ultrasound examination). Furthermore, there was no external reference standard such as MRI available, for logistical reasons.

Although we were pleasantly surprised by the success of our AI approach even on this small training dataset, a limitation of our AI is that it is not fully explainable. The image features that contributed to the diagnosis had not been identified. As future work, we plan to use explainable AI techniques such as a Deep Taylor decomposition to identify regions in the ultrasound image that contributed to a particular diagnosis [18].

## 5. Conclusions

Our data suggests that 3DUS, whether interpreted by human experts or artificial intelligence, is comparable to X-ray in diagnosing pediatric distal radius fractures, with nearly 100% sensitivity, i.e., a negative 3DUS can rule out fracture. 3DUS, particularly when combined with AI, could reduce the need for radiographs for forearm fractures in the emergency department.

## Figures and Tables

**Table 1:** Networks were trained with all combinations of parameters and the top three networks in terms of validation set accuracy were included in the final ensemble model.

Network Parameter	Range of Values
Number of convolutional layers	2–5, step size = 1
Number of fully connected (FC) layers	1–2, step size = 1
Optimizers	Stochastic Gradient Descent (SGD), RMSprop, Adagrad, Adadelta, Adamax ADAM
Dropout	0–50%, step size = 10
Loss Function	Cross-entropy (CE)
Epochs	80

**Table 2:** Statistical analysis of the individual readers and median combination of the individuals.

	J.J.	J.Z.	S.M.	Combined
Sensitivity	1.00 ± 0.00	0.95 ± 0.05	0.95 ± 0.05	1.00 ± 0.00
Specificity	0.83 ± 0.06	0.86 ± 0.06	0.74 ± 0.07	0.90 ± 0.05
Positive predictive value	0.76 ± 0.09	0.78 ± 0.09	0.64 ± 0.09	0.83 ± 0.08
Negative predictive value	1.00 ± 0.00	0.97 ± 0.03	0.97 ± 0.03	1.00 ± 0.00
Positive likelihood ratio	6.00	6.80	3.70	9.75
Negative likelihood ratio	0.00	0.06	0.07	0.00

**Table 3:** Diagnostic accuracy of AI evaluation on individual image slices (per slice), 3DUS sweeps (per sweep), and at the patient level (per patient) in test data not used for AI network training.

Per Image					
Image Type	Normal	Fractured	Sensitivity	Specificity	Accuracy
2D Image Volar Sagittal	504	277	98%	89%	94%
2D Image Dorsal Sagittal	486	373	99%	90%	95%
**Per Sweep**					
Image Type	Normal	Fractured	Sensitivity	Specificity	Accuracy
Sweep Volar Sagittal	23	13	85%	91%	89%
Sweep Dorsal Sagittal	23	13	100%	87%	92%
**Per Patient**					
Image Type	Normal	Fractured	Sensitivity	Specificity	Accuracy
Sweep Volar and Dorsal	23	13	100	87%	92%

## Data Availability

The data presented in this study are available upon request from the corresponding author. The data are not publicly available due to patient privacy requirements of clinical data.
